# Selective Estrogen Receptor Modulators Suppress Hif1α Protein Accumulation in Mouse Osteoclasts

**DOI:** 10.1371/journal.pone.0165922

**Published:** 2016-11-01

**Authors:** Mayu Morita, Yuiko Sato, Ryotaro Iwasaki, Tami Kobayashi, Ryuichi Watanabe, Takatsugu Oike, Kana Miyamoto, Yoshiaki Toyama, Morio Matsumoto, Masaya Nakamura, Hiromasa Kawana, Taneaki Nakagawa, Takeshi Miyamoto

**Affiliations:** 1 Division of Oral and Maxillofacial surgery, Department of Dentistry and Oral Surgery, Keio University School of Medicine, 35 Shinano-machi, Shinjuku-ku, Tokyo 160-8582, Japan; 2 Department of Orthopedic Surgery, Keio University School of Medicine, 35 Shinano-machi, Shinjuku-ku, Tokyo 160-8582, Japan; 3 Department of Musculoskeletal Reconstruction and Regeneration Surgery, Keio University School of Medicine, 35 Shinano-machi, Shinjuku-ku, Tokyo 160-8582, Japan; 4 Department of Advanced Therapy for Musculoskeletal Disorders, Keio University School of Medicine, 35 Shinano-machi, Shinjuku-ku, Tokyo 160-8582, Japan; University of Oulu, FINLAND

## Abstract

Anti-bone resorptive drugs such as bisphosphonates, the anti-RANKL antibody (denosumab), or selective estrogen receptor modulators (SERMs) have been developed to treat osteoporosis. Mechanisms underlying activity of bisphosphonates or denosumab in this context are understood, while it is less clear how SERMs like tamoxifen, raloxifene, or bazedoxifene inhibit bone resorption. Recently, accumulation of hypoxia inducible factor 1 alpha (Hif1α) in osteoclasts was shown to be suppressed by estrogen in normal cells. In addition, osteoclast activation and decreased bone mass seen in estrogen-deficient conditions was found to require Hif1α. Here, we used western blot analysis of cultured osteoclast precursor cells to show that tamoxifen, raloxifene, or bazedoxifene all suppress Hif1α protein accumulation. The effects of each SERM on osteoclast differentiation differed *in vitro*. Our results suggest that interventions such as the SERMs evaluated here could be useful to inhibit Hif1α and osteoclast activity under estrogen-deficient conditions.

## Introduction

Many developing countries show significantly increased numbers of aging individuals, resulting in a sharply increased number of osteoporosis patients and a parallel increase in the number of bone fragile fracture patients [1]. To prevent these conditions, several reagents have been developed such as either anti-bone resorptive or bone-forming drugs [2]. Among these, bisphosphonates, including alendronate and risedronate, the monoclonal antibody demosumab, and selective estrogen receptor modulators (SERMs) are anti-bone resorptive, while teriparatide is categorized as a bone-forming drug [2–4]. Mechanisms underlying bisphosphonate and denosumab activity are well-characterized [5]. For example, bisphosphonates are taken up by osteoclasts upon bone-resorption and inhibit the geranylgeranyl pathway, promoting osteoclast apoptosis [6]. By contrast, denosumab recognizes and inactivates the receptor activator of nuclear factor kappa B ligand (RANKL), blocking osteoclast differentiation and activation [7, 8].

Currently, several SERMs are being utilized clinically [3]. Tamoxifen is used as breast cancer treatment, and it inhibits osteoclast-driven bone resorption [9, 10]. The SERMs raloxifene and bazedoxifene also both reportedly inhibit bone resorptive activity in post-menopausal osteoporosis patients [3, 11–13] and have been used to prevent bone fragility fractures. Binding of SERMs to estrogen receptors (ERs) modulates the receptor’s conformation or ability to form a complex with co-regulators, which in turn, alters their transcriptional activity [14–19]. However, how SERMs inhibit bone resorption mechanistically remains unclear.

Analysis of post-menopausal development of osteoporosis indicates that ERα expressed in osteoclasts function to block osteoclast activation and bone loss [20–22]. Furthermore, our previous analysis of ovariectomized (OVX) mice indicated that hypoxia inducible factor 1 alpha (Hif1α) is required for osteoporosis development under an estrogen-deficient condition [23]. Specifically, Hif1α protein accumulation in osteoclasts was continuously suppressed by estrogen in pre-menopausal estrogen-sufficient conditions but accumulated in osteoclasts in estrogen-deficient conditions [23]. Moreover, treatment of OVX mice with a Hif1α-inhibitor completely abrogated estrogen deficiency-induced osteoclast activation and bone loss [23]. We have also shown that eldecalcitol, a vitamin D analogue and inhibitor of bone resorption in osteoporosis patients, functions as a Hif1α inhibitor [24]. Thus, Hif1α could serve as a therapeutic target to block osteoclast activation and bone loss under estrogen-deficient conditions.

Here, we define mechanisms underlying the anti-bone resorbing function of SERMs. To do so, we treated primary osteoclast precursor cells with SERMs in normal and estrogen-free conditions and evaluated osteoclastogenesis by multi-nuclear osteoclast formation and expression of osteoclastic genes such as *Cathepsin K*, *nuclear factor of activated T cells 1 (NFATc1) and dendritic cell specific transmembrane protein (DC-STAMP)*. In addition, we assessed the effects of SERMs on Hif1α protein levels. We report that although the SERMs tamoxifen, raloxifene and bazedoxifene have varying effects on osteoclast differentiation, they all suppress Hif1α protein accumulation in osteoclasts grown under hypoxic and estrogen-free conditions. Thus, evaluation of Hif1α protein levels in osteoclasts grown under hypoxia *in vitro* may serve as a way to predict whether potential therapies will inhibit osteoclast bone-resorption activity *in vivo*.

## Materials and Methods

### Mice

Wild-type mice on a C57BL/6 background were purchased from Sankyo Labo Service (Tokyo, Japan). Animals were maintained under specific pathogen-free conditions and housed up to 5 mice per cage, and were kept in a 12 h light/dark cycle controlled rooms at the animal facility of the Keio University. Sterile distilled water and standard diet (CLEA Rodent Diet CE-2, Japan) was available *ad libitum*. Animal experiments were reviewed and approved by The Keio University Institutional Animal Care and Use Committee (Permit Number: 09092–14). Animal sacrifice was humanely performed by cervical dislocation for adult mice. This study was specifically approved by the Keio University animal care committee.

### *In vitro* osteoclast formation

Bone marrow cells isolated from femurs and tibias were cultured for 72 h in α-MEM (Sigma-Aldrich Co.) containing 10% fetal bovine serum (FBS) (JRH Biosciences) and GlutaMax (Invitrogen Corp.) or phenol red-free media containing 10% charcoal-stripped FBS (Thermo Fisher Scientific K.K., Yokohama, Japan) supplemented with M-CSF (50 ng/mL, Kyowa Hakko Kirin Co.). After three days of culture, adherent cells were collected and cultured in 96-well plates (1 × 10^5^ cells per well) in the presence or absence of M-CSF (50 ng/mL) and recombinant soluble RANKL (25 ng/mL, PeproTech Ltd.) with or without indicated concentrations of SERMs or estradiol (E2). The medium was replaced every 2 days. Hypoxic culture was performed at 5% O_2_/5% CO_2_ using an INVIVO2 hypoxia workstation (Ruskin Technology Ltd., Bridgend, UK) as previously described [23–26].

Osteoclastogenesis was evaluated by tartrate resistance acid phosphatase (TRAP) staining, and TRAP-positive multi-nuclear cells containing more than three nuclei were scored as osteoclasts [27].

### Quantitative PCR analysis

In three independent analyses, total RNAs were extracted from bone marrow cultures using an RNeasy kit (Qiagen, Venlo, Limburg, The Netherlands). Complementary DNA (cDNA) was prepared by using oligo (dT) primers and reverse transcriptase (Wako Pure Chemicals Industries). Quantitative PCR was performed using SYBR Premix ExTaq II reagent and a DICE Thermal cycler (Takara Bio Inc.), according to the manufacturer’s instructions. *β-actin* expression served as an internal control. Primers for realtime PCR were:

*β-actin*-forward: 5’-TGAGAGGGAAATCGTGCGTGAC-3’

*β-actin*-reverse: 5’-AAGAAGGAAGGCTGGAAAAGAG-3’

*Cathepsin K*-forward: 5’-ACGGAGGCATTGACTCTGAAGATG-3’

*Cathepsin K* -reverse: 5’-GGAAGCACCAACGAGAGGAGAAAT-3’

*NFATc1*-forward: 5’-CAAGTCTCACCACAGGGCTCACTA-3’

*NFATc1*-reverse: 5’-GCGTGAGAGGTTCATTCTCCAAGT-3’

*DC-STAMP*-forward: 5’-TCCTCCATGAACAAACAGTTCCAA-3’

*DC-STAMP*-reverse: 5’-AGACGTGGTTTAGGAATGCAGCTC-3’

### Western blot analysis

Whole cell lysates were prepared from Raw264.7 cell cultures using RIPA buffer (1% Tween 20, 0.1% SDS, 150 mM NaCl, 10 mM Tris-HCl (pH 7.4), 0.25 mM phenylmethylsulfonylfluoride, 10 μg/mL aprotinin, 10 μg/mL leupeptin, 1 mM Na3VO4, 5 mM NaF (Sigma-Aldrich Co.)). Raw264.7 cells are murine leukemic cells transformed by Abelson murine leukemia virus infection [26] that have served as models of osteoclast progenitor cells [28]. Proteins were subjected to SDS-PAGE, transferred to a PVDF membrane (EMD Millipore Corp.), and detected using anti-Hif1α (#NB100-479, 1:1000 dilution; Novus Biologicals, Littleton, CO, USA), and anti-Vinculin (#9131, 1:1000 dilution; Sigma-Aldrich Co.), as previously described [23–25].

### Replication of *in vitro* experiments

At least three independent experiments were performed for all *in vitro* experiments, and representative data are shown.

### Statistical analyses

Statistical analyses were performed using the unpaired two-tailed Student’s *t*-test (*P<0.05; **P<0.01; ***P<0.005; NS, not significant, throughout the paper). All data are shown as means ± SD.

## Results

### Tamoxifen inhibits osteoclast differentiation

**Fig 1:**
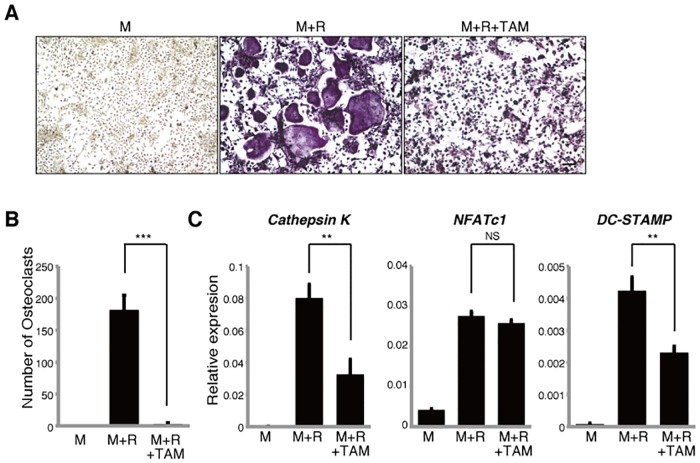
Tamoxifen inhibits osteoclast differentiation *in vitro*. Osteoclast progenitors from wild-type mice were cultured with or without tamoxifen (TAM, 1μM) in the presence or absence of M-CSF 50ng/ml (M) and RANKL 25ng/ml (R). Osteoclast formation was evaluated by TRAP staining (**A**), by the number of multi-nuclear TRAP-positive cells (**B**) and by *Cathepsin K*, *NFATc1*, *DC-STAMP* expression as analyzed by realtime PCR (**C**). Data represent mean *Cathepsin K*, *NFATc1 or DC-STAMP* expression relative to *β-actin* ± SD (*n* = 3). Bar = 100 μm. **P<0.01; ***P<0.001; NS, not significant (unpaired two-tailed Student’s *t*-test). Representative data of at least three independent experiments are shown.

Tamoxifen reportedly inhibits osteoclast bone reorption [10]. To determine if tamoxifen can inhibit osteoclast differentiation *in vitro*, we isolated osteoclast progenitor cells from wild-type mice and cultured them in the presence of M-CSF and RANKL with or without tamoxifen. Osteoclastogenesis was evaluated by assessing formation of multi-nuclear TRAP-positive cells containing more than three nuclei and by expression levels of the osteoclast-related markers *Cathepsin K*, *NFATc1* and *DC-STAMP* (Fig 1). Although mono-nuclear osteoclasts were formed in the presence of tamoxifen, tamoxifen treatment significantly inhibited multi-nuclear osteoclast formation induced by M-CSF and RANKL (Fig 1A and 1B) as well as *Cathepsin K* and *DC-STAMP* expression compared with untreated cells (Fig 1C), suggesting that tamoxifen inhibits osteoclast differentiation.

### Other SERMs have varying effects on osteoclast differentiation

**Fig 2:**
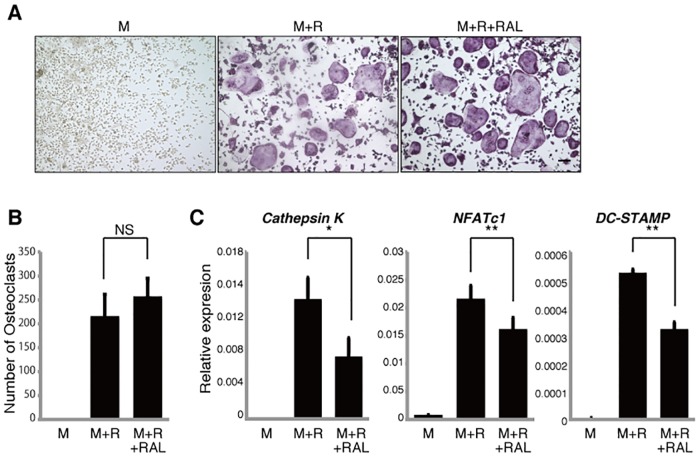
Raloxifene inhibits osteoclastic gene expression *in vitro*. Osteoclast progenitors from wild-type mice were cultured with or without raloxifene (RAL, 1μM) in the presence or absence of M-CSF 50ng/ml (M) and RANKL 25ng/ml (R). Osteoclast formation was evaluated by TRAP staining (**A**), by the number of multi-nuclear TRAP-positive cells (**B**) and by *Cathepsin K*, *NFATc1*, *DC-STAMP* expression as analyzed by realtime PCR (**C**). Data represent mean *Cathepsin K*, *NFATc1 or DC-STAMP* expression relative to *β-actin* ± SD (*n* = 3). Bar = 100 μm. *P<0.05; **P<0.01; NS, not significant (unpaired two-tailed Student’s *t*-test). Representative data of at least three independent experiments are shown.

To determine whether other SERMs have effects comparable to tamoxifen, we first evaluated the effect of raloxifene on differentiation of cultured osteoclast progenitors. Unlike tamoxifen effects, formation of multi-nuclear TRAP-positive osteoclasts was not inhibited by raloxifene (Fig 2A and 2B). Raloxifene treatment did, however, inhibit expression of osteoclastic genes such as *Cathepsin K*, *NFATc1* and *DC-STAMP* in osteoclasts (Fig 2C).

**Fig 3:**
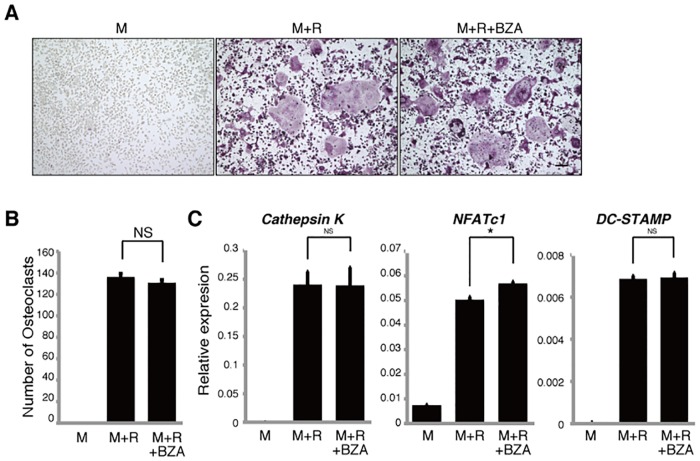
Bazedoxifene does not inhibit osteoclastogenesis *in vitro*. Osteoclast progenitors from wild-type mice were cultured with or without bazedoxifene (BZA, 1μM) in the presence or absence of M-CSF 50ng/ml (M) and RANKL 25ng/ml (R). Osteoclast formation was evaluated by TRAP staining (**A**), by the number of multi-nuclear TRAP-positive cells (**B**) and by *Cathepsin K*, *NFATc1*, *DC-STAMP* expression as analyzed by realtime PCR (**C**). Data represent mean *Cathepsin K*, *NFATc1 or DC-STAMP* expression relative to *β-actin* ± SD (*n* = 3). Bar = 100 μm. *P<0.05; NS, not significant (unpaired two-tailed Student’s *t*-test). Representative data of at least three independent experiments are shown.

We then performed similar tests in osteoclast precursors using the SERM bazedoxifene and found that neither multi-nuclear TRAP-positive osteoclast formation nor osteoclastic gene expression was suppressed (Fig 3A–3C), although bazedoxifene has been shown to inhibit osteoclast bone resorption in post-menopausal osteoporosis patients [29, 30].

### SERMs have varying effects on differentiation of osteoclasts cultured in estrogen-free conditions

**Fig 4:**
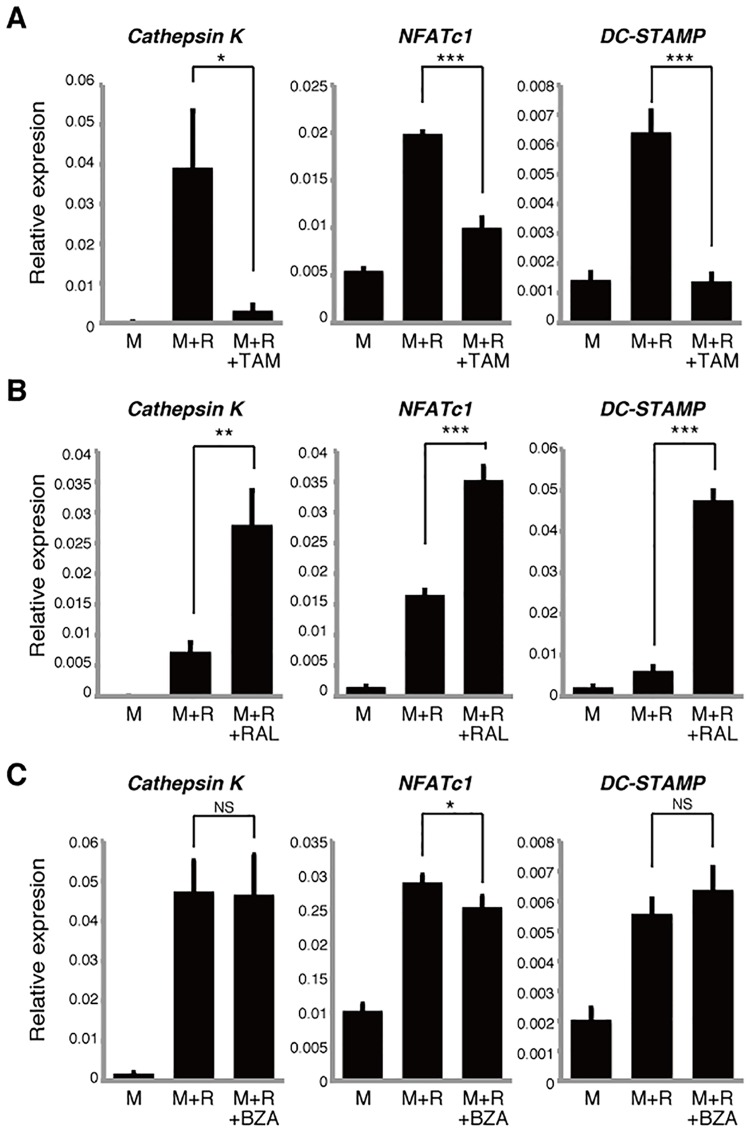
SERM effects on osteoclastogenesis vary in estrogen-free culture conditions. Osteoclast progenitors from wild-type mice were cultured with tamoxifen (TAM, 1μM) (**A**), raloxifene (RAL, 1μM) (**B**) or bazedoxifene (BZA, 1μM) (**C**) in the presence or absence of M-CSF 50ng/ml (M) and RANKL 25ng/ml (R) in phenol red-free medium. *Cathepsin K*, *NFATc1* and *DC-STAMP* expression as analyzed by realtime PCR. Data represent mean *Cathepsin K*, *NFATc1 or DC-STAMP* expression relative to *β-actin* ± SD (*n* = 3). *P<0.05; **P<0.01; ***P<0.001; NS, not significant (unpaired two-tailed Student’s *t*-test). Representative data of at least three independent experiments are shown.

SERMs reportedly act via both ERα-dependent and -independent mechanisms [14, 16]. The presence of phenol red in culture media reportedly promotes estrogenic effects in several cell types; likewise, fetal bovine serum contains estrogen [23]. Thus to assess activity of tamoxifen, raloxifene or bazedoxifene in estrogen-free osteoclast culture conditions, we utilized phenol red-free media and estrogen-depleted serum (Fig 4). Since osteoclast formation promoted by M-CSF and RANKL is strongly inhibited in estrogen-free relative to normal culture conditions, we evaluated osteoclast differentiation based on osteoclastic gene expression rather than multi-nuclear TRAP-positive cell formation. Expression of *Cathepsin K*, *NFATc1* and *DC-STAMP* was significantly inhibited by tamoxifen in estrogen-free conditions (Fig 4A), although *NFATc1* expression was not significantly changed by tamoxifen treatment in normal culture conditions (Fig 1C). Raloxifene treatment significantly elevated *Cathepsin K*, *NFATc1* and *DC-STAMP* expression in osteoclasts grown in estrogen-depleted conditions (Fig 4B), although all three genes had been significantly inhibited in normal culture by comparable treatment (Fig 2C). Moreover, *NFATc1* expression was significantly inhibited by bazedoxifene in estrogen free-conditions (Fig 4C), although expression of *NFATc1* is upregulated by comparable treatment in normal culture conditions (Fig 3C). Overall, despite these variations, the effects of SERMs on osteoclast differentiation in estrogen-free conditions differed from those seen in normal culture conditions.

### Hif1α protein levels in osteoclasts are suppressed by SERMs

**Fig 5:**
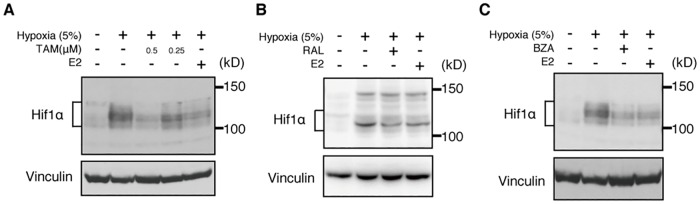
Hif1α protein accumulation is suppressed by SERMs. Western blot analysis of Raw264.7 cells cultured in hypoxic conditions with or without tamoxifen (TAM, 0.5μM, 0.25μM) (A), raloxifene (RAL, 1μM) (B), or bazedoxifene (BZA, 1μM) (C) or estradiol (E2, 1μM). Vinculin expression serves as an internal control. In all panels, –(minus) symbols in the hypoxia row indicate normoxic conditions. Representative data of at least three independent experiments are shown.

Finally, given that Hif1α protein is reportedly a target of estrogen [23], we asked whether Hif1α protein levels in osteoclasts decrease following SERM treatment (Fig 5). To do so, we cultured Raw264.7 osteoclast progenitor cells in the presence of RANKL with or without SERMs in normoxic or hypoxic conditions, and evaluated Hif1α protein levels by western blots (Fig 5A–5C). We did not detect Hif1α protein in normoxic conditions, but Hif1α protein accumulated in hypoxic conditions, and that accumulation was suppressed by treatment with tamoxifen, raloxifene or bazedoxifene or by estrogen (E2) (Fig 5A–5C).

## Discussion

**Fig 6:**
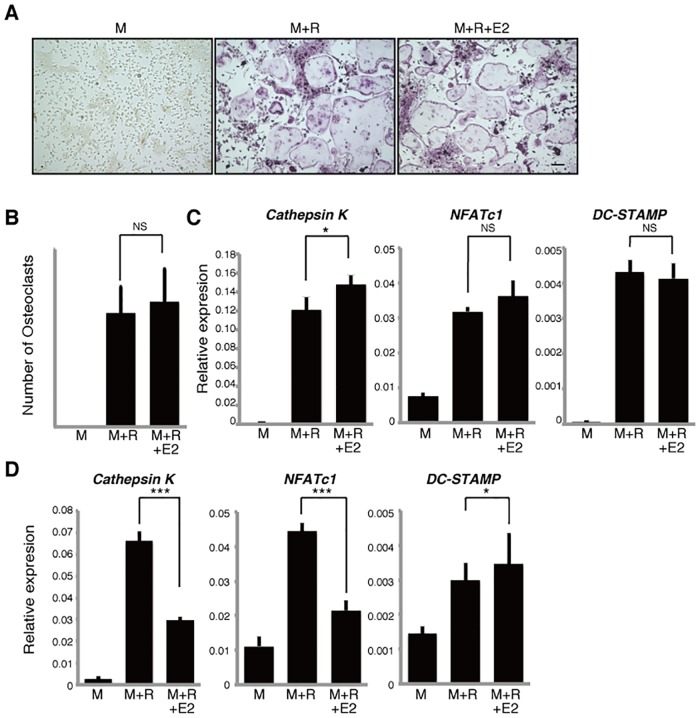
Effects of estradiol on osteoclastogenesis in normal and estrogen-free conditions. Osteoclast progenitors from wild-type mice were cultured with or without estradiol (E2, 1μM) in the presence or absence of M-CSF 50ng/ml (M) and RANKL 25ng/ml (R). Osteoclast formation was evaluated by TRAP staining (**A**), by the number of multi-nuclear TRAP-positive cells (**B**) and by *Cathepsin K*, *NFATc1*, *DC-STAMP* expression as analyzed by realtime PCR in normal (**C**) or estrogen-free (**D**) medium. Data represent mean *Cathepsin K*, *NFATc1 or DC-STAMP* expression relative to *β-actin* ± SD (*n* = 3). Bar = 100 μm. *P<0.05; ***P<0.001; NS, not significant (unpaired two-tailed Student’s *t*-test). Representative data of at least three independent experiments are shown.

Prevention of bone fragility fractures and control of osteoporosis are global health issues in developed countries. To date, most reagents used to prevent bone fragility fractures in osteoporosis patients are anti-bone resorptive (among them SERMs), although a few activate bone formation. Mechanisms underlying SERM activity in this context remain unclear. Under sex hormone-depleted conditions, Hif1α was demonstrated to be a therapeutic target in conditions of post-menopausal [23] and male [25] osteoporosis. SERM treatment also inhibits osteoclast bone resorption in post-menopausal osteoporosis patients [9, 11, 13, 31]. To date, there has been no definitive mechanistic explanation for why SERMs act as anti-bone resorptive agents. Our finding that SERM treatment blocks Hif1α accumulation provides a possible explanation for this outcome. Our observations also imply that the fact that the effects of any drugs on osteoclast differentiation *in vitro* is not sufficient to suggest that that drug would have anti-bone resorptive effects *in vivo* or in patients, as the three drugs tested here had different effects on *in vitro* osteoclastogenesis, although all of the SERMs tested were reportedly acting as inhibitors of osteoclast bone resorption [3, 4, 9, 11, 13], but all blocked Hif1α accumulation in osteoclasts. More studies are required to determine whether tamoxifen alters activities other than Hif1α protein suppression in osteoclasts. Moreover, the effects of SERMs on osteoclastic gene expression differed in normal and estrogen-free conditions, an outcome that we cannot yet explain. However, in normal conditions, following addition of estradiol to cultures of osteoclast precursors, we found that only *Cathepsin K*, but not *NFATc1* and *DC-STAMP*, expression was upregulated in osteoclasts and multi-nuclear osteoclast formation was not accelerated (Fig 6A–6C). By contrast, in estrogen-free conditions *Cathepsin K* and *NFATc1* expression was significantly inhibited by estradiol (Fig 6D). Thus, none of the SERMs tested here recapitulate the effects of estradiol *in vitro* in terms of osteoclastic gene expression. Since estradiol reportedly suppresses Hif1α protein accumulation in osteoclasts grown in hypoxic, estrogen-free conditions [23], suppression of Hif1α protein in osteoclasts is an activity shared by both SERMs and estradiol *in vitro*.

Since post-menopausal, estrogen-deficient conditions promote osteoclast activation leading to bone loss, estrogen administration is considered a means to reverse these conditions. Indeed, hormone replacement therapy (HRT) increases bone mass in post-menopausal osteoporosis patients, although treatment can have adverse effects [32]. Other agents such as bisphosphonates and denosumab can block osteoclastic bone resorption in osteoporosis patients, but these, too, can have adverse effects such as osteonecrosis of the jaws (ONJ) and severely suppressed bone turnover (SSBT) [33–35]. Comparable adverse effects have not been reported in patients treated with SERMs [3, 31]. However, SERMs have been found to be less effective in inhibiting osteoclastic bone resorption than bisphosphonates [4, 36]. This observation may support the idea that SERMs are Hif1α inhibitors, since treatment of normal mice with Hif1α inhibitors does not block physiological osteoclast activity in estrogen- or testosterone-sufficient conditions [23, 25]. In contrast, administration of bisphosphonates or an anti-RANKL antibody to comparable, hormone-sufficient mice strongly inhibits physiological osteoclast activity, increasing bone mass [37].

Unlike individuals treated with bisphosphonates or denosumab, patients treated with HRT and SERMs can exhibit thrombosis development in deep veins [38–40], although the cause of these complications remains unclear. ERα is reportedly required to suppress Hif1α protein in osteoclasts [23], while SERMs are thought to act via ERα-dependent or independent mechanism [14–16]. Hif1α has also been considered a therapeutic target in some malignancies [41]. Tamoxifen and raloxifene are effective in inhibiting tumor burden in some breast cancers, while HRT promotes tumor development [42]. Further studies are needed to clarify molecular mechanisms underlying thrombosis and tumor development by HRT and SERMs.

Estrogen also inhibits osteoclast activation indirectly via osteoblasts [43–45]. Moreover, osteoclast-specific ERα conditional knockout (ERα cKO) mice exhibit reduced bone mass relative to controls, and OVX does not decrease bone mass in ERα cKO mice [20], suggesting that ERα expressed in osteoclasts plays a crucial role in regulating bone mass.

We previously demonstrated that Hif1α could be a therapeutic target in osteoporosis [23], leading us to test the effects of candidate anti-bone resorptive agents on Hif1α protein suppression in osteoclasts *in vitro*. Eldecalcitol, which is used to inhibit bone resorption in osteoporosis patients [46], lowers Hif1α protein levels in osteoclasts [24], comparable to our observation following SERM treatment. To date, anti-osteoporosis agents have been tested in animal models such as OVX mice *in vivo*, but this system does not allow efficient testing of numerous candidate agents due to costs and time frame of testing. Screening *in vitro* for Hif1α protein suppression in osteoclasts could substitute for animal models and reduce expenses and time required to evaluate anti-bone resorbing agents.

## Conclusions

SERMs act as inhibitors of Hif1α, a therapeutic target of post-menopausal osteoporosis, in osteoclasts under an estrogen-deficient condition. Testing inhibitory effects on Hif1α protein in osteoclasts *in vitro* is useful to screen candidate anti-bone resorptive agents before animal models.
